# Family Satisfaction With Critical Care: Before and After the COVID-19 Outbreak

**DOI:** 10.7759/cureus.33853

**Published:** 2023-01-16

**Authors:** Núria Jorge, Isabel Hipólito-Reis, Nuno Esteves, Liliana Costa, Inês Mendonça, Teresa Oliveira, José Paiva

**Affiliations:** 1 Intensive Care Medicine Department, Centro Hospitalar Universitário de São João, Porto, PRT

**Keywords:** decision-making, quality improvement, covid-19, patient care, family, critical care

## Abstract

Introduction

Family satisfaction with intensive care units (ICU) is recognized as a key component of the quality of care. As a result, family members are now more involved in the care process, and their needs are recognized throughout the ICU stay. The coronavirus disease 2019 (COVID-19) changed healthcare worldwide, due to the several restrictions imposed; the communication patterns changed drastically, and institutions were forced to adapt to create a balance between security and the needs of relatives. The aim of this study was to assess family members' satisfaction with the ICU and determine if the COVID-19 restructuring affected family satisfaction.

Methods

A prospective observational study was performed among the designated family members (DFM) of ICU patients over two time periods, a pre-pandemic period from December 2019 to February 2020 and a pandemic period from May 2020 to February 2021. The Family Satisfaction in the Intensive Care Unit 24 (FS-ICU 24) questionnaire, which was given to the DFM, was the instrument used to determine family satisfaction.

Results

The study involved 290 DFM, 175 during the pre-pandemic phase and 115 during the pandemic period. The overall and domain-specific family satisfaction scores were high (score > 80) in both the pre-pandemic and pandemic periods. The greatest satisfaction levels were related with symptom management and how nurses and doctors cared for the patient. No statistical differences were found between the two time periods. Lastly, a positive association between the two domains explored by FS-ICU 24, satisfaction with care and satisfaction with decision-making process, was verified in both time frames.

Conclusion

The data obtained revealed very good outcomes on the different FS-ICU 24 domains, in line with other studies in literature. No significant differences were found between the pre-pandemic and pandemic periods, suggesting that the measures implemented during the COVID-19 were successful. The importance of involving families in the decision-making process, providing them with accurate information, and active listening, as well as using better communication skills, is emphasized throughout all these results. The relevance of measuring family satisfaction should be brought to the attention of family members and healthcare professionals so that additional research may be conducted.

## Introduction

The key outcomes of significant randomized controlled trials in intensive care medicine are disease-focused [1]. In fact, significant clinical advances over the past few decades have enabled the lowering of mortality in critically ill patients, which is highly important for intensivists [1]. However, patient-centered outcomes have grown in importance over the years, and the quality of life and functional, cognitive, and neurological performance stand out in the scope of action of critical care physicians [1]. Family members and caregivers are crucial components for enhancing these patient-centered outcomes.

Hospitalization in the intensive care unit (ICU) is a potentially challenging and traumatic experience for patients and their families. The awareness of health professionals for this reality has created a propensity for change, with a focus not only on the patient but also on their caregivers [2,3]. There seems to be an impending paradigm shift, in which the family is involved in the care process and their needs are recognized and valued throughout the ICU stay.

The term "post-intensive care syndrome" refers to the residual disability of ICU survivors, which can manifest as physical, psychological, or cognitive sequelae [4]. Family members of these patients may experience a cluster of mental complications, such as major depression or posttraumatic stress disorder, collectively known as post-intensive care syndrome family (PICS-F) [4,5]. The assessment of the family's needs and degree of satisfaction is therefore essential [6]. Since 1970, several questionnaires have been designed to assess family needs and satisfaction. The "Family Satisfaction in the Intensive Care Unit 24" (FS-ICU 24) is the most widely used and validated instrument, namely, for psychometric properties [7,8].

Satisfaction is by itself a marker of the quality of care, and the perspectives of family members, as well as the patient's perceptions, have been highlighted and used as one of the quality indicators in intensive care medicine [9,10]. On the other hand, this assessment provides information about the values and expectations of the patients and their families [10], allowing health professionals to adapt the care provided.

Health professionals' proactive communication stands out among several recognized interventions and plays an important role in the care process and satisfaction associated with it [11]. The coronavirus disease 2019 (COVID-19) created enormous pressure on hospitals, which led to the adoption of mandatory distancing and social isolation measures in most European countries, including Portugal [12,13]. This health crisis resulted in a dramatic shift in communication patterns involving patients and their families, particularly in the ICU setting, where nonverbal communication such as the tone of voice, posture, or facial expression were lost [8].

Institutions have adapted to this reality, creating different strategies to find a balance between the need for safety and the needs of the family. In this article, the authors present data of family satisfaction, regarding ICU care and communication strategies, before and during the COVID-19 pandemic. The aim of this study was to assess family members' satisfaction with the ICU and determine if the COVID-19 restructuring affected family satisfaction.

## Materials and methods

Design

A prospective observational study was performed among family members of ICU patients, over two time periods, from December 2019 to February 2020 (pre-pandemic period) and from May 2020 to February 2021 (pandemic period).

Setting and participants

The study was conducted in the intensive care department of a tertiary hospital in northern Portugal. The pre-pandemic ICU policy included a daily visiting window (4 pm to 9 pm), as well as a defined protocol for welcoming relatives in the ICU. However, there were no written instructions on how to take care of the family members during the ICU stay. The clinical updates were given daily by the medical team in charge. The prohibition of hospital visits was implemented between March and June 2020 as a result of the COVID-19 pandemic, in compliance with regional and national legislation. However, for critically ill patients, short visits of one person were exceptionally allowed during this period. After that, local policies were softened to shorter visit schedules for ICU patients after five days of hospitalization. These local policies were in effect until the study's conclusion.

Due to the implemented restrictions, several strategies were devised to bridge the gap between patients and family members. Senior nurses were in charge of reaching out to families by phone call or videophone call twice daily to keep them informed of their relative's clinical condition, and every day, families were signaled for extra contact by the medical team. Furthermore, all eligible patients (awake and able to interact) were encouraged to have a daily videoconference call with their relatives.

Table 1 summarizes the differences in protocol/policy between the two time periods analyzed.

**Table 1:** Differences in protocol/policy between the pre-pandemic and pandemic periods ICU: intensive care unit

Differences in protocol/policy between the pre-pandemic and pandemic periods
Pre-pandemic period
Multidisciplinary meeting with the ICU care team held on the first visit
Daily visits were allowed from 4 pm to 9 pm
Daily contact with the healthcare staff during the daily visit
Pandemic period
The ICU physician contacted the family by phone on the admission day
March to June 2020: there were only allowed visits in end-of-life situations
From June 2020: the visits were scheduled in advance and were only allowed for patients admitted for more than five days in the ICU
Senior nurses were responsible for reaching out to families by phone or video call twice daily
Medical contact was made by phone when the patient's condition changed, the nursing staff signaled for extra contact, or the family requested it
Patients that were awake and able to interact were encouraged to have a daily video call with their relatives

A relative is nominated as the designated family member (DFM) upon the patient's admission to the ICU. The designated family member is the person with the closest family, social, and emotional relationship with the patient, by consanguinity and also by intimacy [14], ideally defined by the patient or, if this is not possible, through assessment by the clinical staff. The participants of the study were the DFM; other inclusion criteria included are age above 18, being literate, patient hospitalization of at least 48 hours, and having at least one presential visit to the ICU. Exclusion criteria were relatives who had previously responded to the survey, relatives with no significant relationship with the patient, patients readmitted to the ICU, and family members of patients who died while hospitalized.

The FS-ICU 24 questionnaire and the information letter were delivered on paper to the designated relative between the fourth and sixth day of hospitalization, and in the pandemic period, these documents were given after the first family visit to the ICU. No identifying information was included in the questionnaires, so family members could remain anonymous. The participants were requested to complete the questionnaire and either drop it off in a secure box located in the waiting area or mail it back to us anonymously. Returning questionnaires constituted written informed consent. The study was approved by the local ethics committee (Comissão de Ética do Centro Hospitalar de São João/Faculdade de Medicina da Universidade do Porto) under identification number 322/2021.

Questionnaire

The original FS-ICU 24 was generated from research on the needs of critically ill families, literature on family satisfaction, already-validated satisfaction surveys, conceptual frameworks for patient satisfaction, and a pilot study [15]. The FS-ICU 24 is a 24-item version of the questionnaire that has been refined and condensed [16]. Nowadays, it has been translated to several languages and used in numerous international research studies [3,8,10,14].

FS-ICU 24 questionnaire measures family satisfaction in two domains regarding satisfaction in relation to care and satisfaction in relation to the decision process. The survey also includes demographic inquiries and three open-ended questions where family members can provide free-text comments. Data was collected using the validated FS-ICU 24 version for Portuguese speakers [17].

Statistical analysis

Data analysis was performed using Statistical Package for Social Sciences (SPSS) version 27 (IBM SPSS Statistics, Armonk, NY). The reliability of the Portuguese version of FS-ICU 24 was determined by the assessment of its internal consistency with Cronbach's alpha reliability coefficients in the two analyzed moments.

The collected data was subjected to a descriptive analysis. In order to perform the necessary statistical analysis and as suggested by the survey authors, the questionnaire responses' format (Likert scale) had to be converted to a numerical scale of 0-100, where 0 = poor, 25 = fair, 50 = good, 75 = very good, and 100 = excellent [14]. Demographic information and satisfaction scores were presented as mean and standard deviation (SD) or percentage if appropriate. Considering the two data collection moments, the t-test was utilized to determine whether there were statistically significant differences in the FS-ICU 24's satisfaction items.

Pearson's correlation coefficient was used to test whether there was a statistically significant association between the domains of the FS-ICU 24 instrument, satisfaction with care and satisfaction with the decision-making process, in the two data collection moments. All reported p values are two-tailed, and statistical significance is represented by a p value < 0.05.

## Results

A total of 290 DFM were included: 175 participants were enrolled during the pre-pandemic period and 115 in the second period of data collection. Table 2 summarizes the sociodemographic characteristics of the participants.

**Table 2:** Sociodemographic characteristics of the participants SD, standard deviation; ICU, intensive care unit

Sociodemographic characteristics of participants	Pre-pandemic	Pandemic
Mean age (SD)	47.6 (14.8)	47.8 (15.7)
Gender, n (%)		
Female	105 (60.0)	58 (50.4)
Male	54 (30.9)	48 (41.7)
No answer	16 (9.1)	9 (7.8)
Relationship to patient, n (%)		
Daughter or son	57 (32.6)	32 (27.8)
Spouse	60 (34.3)	31 (27.0)
Parent	14 (8.0)	19 (16.5)
Others	16 (9.1)	21 (18.3)
No answer	19 (10.9)	12 (10.4)
Level of education, n (%)		
Basic (4-6 years)	2 (1.2)	8 (7.0)
Lower secondary (9 years)	18 (10.3)	25 (21.7)
Upper secondary (12 years)	36 (20.6)	38 (33.0)
University	26 (14.9)	31 (27.0)
No answer	93 (53.1)	13 (11.3)
Lives with patient before admission, n (%)		
Yes	94 (53.7)	51 (44.3)
No	61 (34.9)	51 (44.3)
No answer	20 (11.4)	13 (11.3)
If not cohabitant, frequency of seeing the patient before admission, n (%)		
Yearly	1 (0.6)	3 (2.6)
Monthly	3 (1.7)	5 (4.3)
Weekly	15 (8.6)	18 (15.7)
More than once a week	40 (22.9)	23 (20.0)
No answer	116 (66.3)	66 (57.4)
Lives in the same city of the hospital, n (%)		
Yes	38 (21.7)	17 (14.8)
No	119 (68.0)	87 (75.7)
No answer	18 (10.3)	11 (9.6)
Prior experience as a family member of a patient admitted to an ICU, n (%)		
Yes	52 (29.7)	28 (24.3)
No	107 (61.1)	74 (64.3)
No answer	16 (9.1)	13 (11.3)

The description is made in terms of age, gender, relationship to patient, level of education, frequency of contact with the patient, and whether the family member had already had a relative in an ICU before. The mean age of survey respondents in the first and second periods was almost equal, 47.6 (SD = 14.8) and 47.8 (SD = 15.7) years, respectively. The majority of the respondents were female (60.0% and 50.4%), and the most frequent kinship was spouse (34.3% and 27.0%) and daughter/son (32.6% and 27.8%). The vast majority of patients did not reside near the hospital (68.0% and 75.7%). Only a minority had higher education level (14.9% and 27.0%), and only a few participants had a previous experience with a family member admitted to an ICU (29.7% and 24.3%).

Regarding the general aspects of care (Table 3), most DFM were globally satisfied. DFM reported the greatest satisfaction levels with symptom management and with how nurses and doctors cared for the patient. Despite the overall good results, satisfaction was lower regarding the atmosphere in the waiting room item (mean: 75.0 and 76.2) in both moments of the evaluation, which targets an area that may possibly be improved.

**Table 3:** Family satisfaction with care and decision-making SD, standard deviation; ICU, intensive care unit; FS-ICU 24, Family Satisfaction in the Intensive Care Unit 24

Family satisfaction with care in the intensive care unit: FS-ICU 24	Pre-pandemic	Pandemic
	Mean (SD)	Mean (SD)
Family satisfaction with care		
1. How satisfied are you with the courtesy, respect, and compassion your family member (the patient) was given?	91.4 (14.1)	91.4 (15.3)
2a. How well the ICU staff assessed and treated your family member's pain?	92.2 (12.8)	91.0 (15.6)
2b. How well the ICU staff assessed and treated your family member's breathlessness?	92.0 (14.6)	91.8 (14.5)
2c. How well the ICU staff assessed and treated your family member's agitation?	90.0 (15.0)	91.4 (15.9)
3. How satisfied are you with how well the ICU staff showed an interest in your needs?	89.0 (14.7)	87.0 (19.8)
4. How satisfied are you with how well the ICU staff provided emotional support to you?	85.4 (17.1)	87.0 (19.9)
5. How satisfied are you with the teamwork of all the ICU staff that took care of your family member?	90.6 (14.4)	90.0 (16.0)
6. How satisfied are you with the courtesy, respect, and compassion you were given?	88.8 (15.1)	89.0 (16.9)
7. How satisfied are you with how well the nurses cared for your family member?	92.0 (12.8)	92.0 (14.4)
8. How satisfied are you with how often nurses communicated to you about your family member's condition?	89.6 (14.6)	87.0 (20.4)
9. How satisfied are you with how well doctors cared for your family member?	92.0 (14.2)	91.0 (15.9)
10. How satisfied are you with the atmosphere (mood) in the ICU waiting room?	75.0 (20.5)	76.2 (21.6)
11. How satisfied are you with the atmosphere (mood) of the ICU?	83.4 (17.7)	83.4 (17.7)
12. How satisfied are you with your participation in daily rounds?	87.6 (18.7)	85.8 (17.5)
13. How satisfied are you with your participation in the care of your critically ill family member?	87.2 (15.6)	86.4 (18.5)
14. How satisfied are you with the level or amount of healthcare your family member received in the ICU?	89.2 (15.8)	89.2 (16.3)
Family satisfaction with decision-making around the care of critically ill patients		
15. How satisfied are you with how often doctors communicated to you about your family member's condition?	84.4 (16.6)	84.0 (21.1)
16. How satisfied are you with the willingness of the ICU staff to answer your questions?	86.4 (18.3)	85.2 (20.4)
17. How satisfied are you with how well the ICU staff provided you with explanations that you understood?	88.2 (15.2)	86.6 (19.6)
18. How satisfied are you with the honesty of information provided to you about your family member's condition?	89.4 (14.6)	87.6 (18.8)
19. How satisfied are you with how well the ICU staff informed you what was happening to your family member and why things were being done?	87.6 (16.1)	85.0 (21.8)
20. How satisfied are you with the consistency of information provided to you about your family member's condition?	87.0 (16.9)	85.0 (21.7)
21. How satisfied are you with the inclusion in decision-making?	78.8 (22.2)	79.6 (24.5)
22. How satisfied are you with the support during decision-making?	77.4 (21.9)	82.0 (20.6)
23. How satisfied are you with the control over the care?	75.2 (20.9)	78.2 (25.7)
24. How satisfied are you with the time to address concerns and questions when making decisions?	76.2 (19.4)	75.6 (23.2)

Regarding the aspect of decision-making, only approximately 75% were completely satisfied. The scores were particularly low in the items "How satisfied are you with the control over the care?" (mean: 75.2 and 78.2) and "How satisfied are you with the time to address concerns and questions when making decisions?" (mean: 76.2 and 75.6).

No statistically significant differences were found in the different items of the FS-ICU 24 between the two considered periods. Table 4 shows the results regarding the internal consistency of the instrument used in this study (FS-ICU 24) in both moments of data collection. It is possible to conclude that there is an elevated internal consistency in both domains on the two periods of evaluation.

**Table 4:** Internal consistency of the FS-ICU 24 domains FS-ICU 24: Family Satisfaction in the Intensive Care Unit 24

Domain	Cronbach's alpha
Satisfaction with care	
Pre-pandemic	0.922
Pandemic	0.963
Satisfaction with the decision-making process	
Pre-pandemic	0.943
Pandemic	0.946

Table 5 presents the mean score of the questionnaire and of the two domains that are included, at both data collection periods. In both the pre-pandemic and pandemic periods, domain-specific family satisfaction scores were high. The satisfaction with care domain had a mean score of 88.5 and 88.1, respectively, and the satisfaction with the decision-making process domain score was 83.1 in the pre-pandemic period and 82.9 in the pandemic moment.

**Table 5:** Overall FS-ICU 24 scores SD, standard deviation; FS-ICU 24, Family Satisfaction in the Intensive Care Unit 24

Overall FS-ICU 24 scores	Pre-pandemic	Pandemic
Satisfaction with care domain score, mean (SD)	88.5 (4.2)	88.1 (3.9)
Satisfaction with the decision-making process domain score, mean (SD)	83.1 (5.2)	82.9 (3.7)
FS-ICU 24 score, mean (SD)	86.4 (5.4)	86.1 (4.7)

A positive, moderate, and statistically significant association between the two domains was equally verified in the pre-pandemic moment (r = 0.816; p < 0.001) and in the pandemic moment (r = 0.874; p < 0.001), suggesting that the greater the participants' satisfaction with assistance, the greater their satisfaction with the decision-making process.

## Discussion

To the best of our knowledge, this is one of the first studies to compare family satisfaction with the ICU, using a validated tool, in two different periods of time, namely, pandemic and pre-pandemic. Family satisfaction with the ICU was found to be high, and no significant differences were found between the pre-pandemic and pandemic periods. On the other hand, the analysis of the questionnaire item's satisfaction scores revealed potential areas for improvement.

The COVID-19 period brought major changes in ICU logistics. In the pandemic period, family visits were restricted throughout the hospital, raising concerns about communication problems and information inconsistencies between clinicians and families. Determining how the pandemic affected family satisfaction was of paramount importance.

Family satisfaction data had to be collected in a reproducible and validated way. In a systematic review of 27 different tools used to measure family satisfaction, the FS-ICU 24 was found to be one of the most reliable and valid in terms of psychometric properties [7], and it is now the most widely validated tool in this field [14]. In this study and in line with previous research [18], the FS-ICU 24 exhibited very good internal consistency reliability (Cronbach's alpha > 0.90).

The sociodemographic characteristics of the DFM revealed that the populations of the two time periods were similar, allowing a feasible comparison between the two time frames. The item "Where do you live?" shows that the majority of the surveyed lived away from the hospital (68.0% in the pre-pandemic and 75.7% in the pandemic period), reflecting the hospital's status as a reference center for patients from a wide geographic area (northern Portugal), particularly those who require specialized care, such as neurocritical care or extracorporeal membrane oxygenation support.

Interestingly, no statistical differences were found between pre-pandemic and pandemic family satisfaction, measured by the questionnaire. This may be explained by the procedures implemented during the pandemic period to meet the family's needs. We can hypothesize that the strategies mentioned were sufficient to maintain family satisfaction in this period.

Moreover, by the analysis of the overall satisfaction score, we could infer that families are highly satisfied (satisfaction scores > 80) with the care given and with the decision-making process regarding their relatives in this ICU, even in the pandemic period, which is in line with other studies in the literature [3,8,19].

Nevertheless, there are some items that had a lower incidence of "excellent" response (< 80), which must be seen as opportunities for improvement. This occurs in the "satisfaction with care" domain, with the satisfaction with the atmosphere in the ICU waiting room (mean: 75.0 and 76.2), and in the "satisfaction with the decision-making process" domain, with the satisfaction with the inclusion (mean: 78.8 and 79.6), the support (mean: 77.4 and 82.0), the control over care (mean: 75.2 and 78.2), and the time to address concerns and questions (mean: 76.2 and 75.6). Interestingly, these results are in agreement with previous research [3,18].

A moderate and statistically significant positive association (r = 0.816; p < 0.001) between the two domains, satisfaction with care and satisfaction with decision-making process, in the pre-pandemic period suggests that the higher the satisfaction with care, the higher the satisfaction with the decision-making process. The same was also true for the pandemic group (r = 0.874; p <0.001). These findings are consistent with those of Epstein et al. that while studying family satisfaction in a pediatric intensive care unit using FS-ICU 24 demonstrated that care and medical decision-making domains were highly correlated [18]. More research is needed to explain the association between these two domains, but we may hypothesize that a family who feels more engaged in patient care also feels more included in the decision process.

This study had several limitations. One is the different duration of the periods compared, with the pre-pandemic phase lasting three months and the pandemic phase lasting 10 months. This was due to the difficulty to reach out family members who met the inclusion criteria, given all the limitations imposed by COVID-19 safety restrictions, and the excessive workload on the professionals. The 10-month time frame may have given professionals time to adjust to the pandemic-imposed reality. On the other hand, a response bias could not be completely discarded since the response rate could not be determined and relatives who were dissatisfied with the ICU might not have taken part in the survey. The difficulty in enrolling family members can be explained by the lack of human resources and high work burden, as there was no dedicated workforce for this research. However, a lack of interest on the part of healthcare workers and family members, due to a lack of awareness of the importance of the family well-being for patient's care, may also be a contributor. Lastly, although family satisfaction in end-of-life scenarios has been identified as an area for quality improvement in the ICU setting, we restrict the scope of the current study to ICU survivors.

The FS-ICU 24 proved to be a consistent instrument for measuring family satisfaction in our ICU setting, but drawing conclusions from a single research approach requires caution, and further research must be conducted.

## Conclusions

Family satisfaction in critical care scenarios has become a crucial and relevant component of quality assessment in intensive care medicine, as well as a valuable outcome measurement in healthcare. This is one of the first studies comparing family satisfaction in the same ICU before and after the breakthrough of the COVID-19 pandemic.

The data obtained revealed very good outcomes on the different FS-ICU 24 domains, in line with other studies in the literature. No statistical differences were found between the two time periods, suggesting that the measures implemented during the COVID-19 for family communication may have been successful. Additionally, a positive association between the two domains, satisfaction with care and satisfaction with decision-making process, was verified in both time frames, suggesting that the greater the family satisfaction with assistance, the greater the satisfaction with the decision-making process. It is essential to convey to family members and health workers the relevance and value of measuring family satisfaction.
